# Children’s, parents’ and health professionals’ views on the management of childhood asthma: a qualitative study

**DOI:** 10.1038/s41533-017-0053-7

**Published:** 2017-09-11

**Authors:** Aidan Searle, Russell Jago, John Henderson, Katrina M. Turner

**Affiliations:** 1 National Institute for Health Research (NIHR) Biomedical Research Centre (Nutrition, Diet and Lifestyle theme) at the University Hospitals Bristol NHS Foundation Trust and the University of Bristol, Bristol, BS2 8AE UK; 2 Centre for Exercise, Nutrition & Health Sciences, School for Policy Studies, University of Bristol, 8 Priory Road, Bristol, BS8 1TZ UK; 3 The National Institute for Health Research Collaboration for Leadership in Applied Health Research and Care West (NIHR CLAHRC West) at University Hospitals Bristol NHS Foundation Trust, Bristol, UK; 4 School of Social & Community Medicine, University of Bristol, Oakfield House, Oakfield Grove, Clifton, Bristol, UK; 5 Centre for Academic Primary Care, School of Social & Community Medicine, University of Bristol, Canynge Hall, 39 Whatley Road, Bristol, UK

## Abstract

The management of childhood asthma is often sub-optimal. Parents and other caregivers are primarily responsible for disease management and this responsibility includes communication with health professionals. The aim of this multi-perspective qualitative study was to explore the views of children, parents and health professionals to gain insight into the approach to clinical care in the management of childhood asthma. Interviews were held with nine parent–child (6–8 years) dyads, and 13 health professionals working in primary and secondary care. Interviews were transcribed verbatim and analysed thematically. Three key themes emerged that were common to all data sets; (1) Child and parent awareness of symptoms; (2) Management and child wellbeing; and (3) Professional communication education and consultation with families. Although some children demonstrate good awareness of symptoms and appropriate use of medication, some parents expressed difficulty in identifying triggers and symptoms of asthma. Furthermore, parents lacked awareness regarding appropriate use of medication for preventing and managing symptoms of asthma. Health professionals believed that communication and education was lacking. Data from all participants suggested that consultations could be enhanced with greater emphasis on children’s and parents’ perceptions of asthma in the development of asthma management plans.

## Childhood asthma: Guiding families through disease management

Both parents’ and children’s perceptions and understanding of childhood asthma should be considered when developing asthma management plans. The management of asthma is challenging and can result in poor disease outcomes if care is not taken. An individual’s perception of their (or their child’s) asthma can also affect the efficacy of treatment. Aidan Searle at the Bristol Biomedical Research Centre, UK, and co-workers, interviewed nine parent-child groups and thirteen health professionals to determine their perceptions of childhood asthma management in primary care. While some children had a strong awareness of symptoms and appropriate medication use, some parents found it difficult to identify asthma triggers and symptoms. Parents also displayed a lack of understanding of management through medication. Health professionals focused on the need for clearer information for families when guiding management of childhood asthma.

## Introduction

Over one million (1 in 11) children in the UK are currently receiving treatment for asthma and the majority of these patients are managed exclusively in primary care.^1, 2^

During early childhood, parents and other caregivers are primarily responsible for disease management. This responsibility includes communication with health professionals, medication administration, identification and avoidance of triggers, setting medical appointments, and obtaining prescriptions.^3^ Sub optimal adherence to daily medication is the commonest cause of poor symptom control in childhood asthma and may lead to increased asthma morbidity,^4^ poor school attendance, adverse effects on family life, and increased healthcare costs.^5^

There are circumstances that compromise asthma management, e.g., children and carers may not remember to administer medication and children may be responsible for administering their own medication at school.^6–8^ Difficulties with inhalation are more likely to be experienced by children than by adults and even if children can demonstrate good technique in clinic, they are often unable to use inhalers competently outside of the clinic due to forgetting instructions or choosing a different technique due to ease or social constraints.^9, 10^ The impact of non-adherence depends on the severity of the condition and the effectiveness of the treatment. However, so called ‘intelligent non-adherence’ may also be an issue if the treatment is deemed ineffective or harmful.^11, 12^ Thus, parents and children must accept the diagnosis and agree that regular preventative medication is necessary to effectively manage asthma. The overall aim of childhood asthma management is to control symptoms allowing the child to lead a normal active life.^1, 2^

Studies exploring adherence in childhood asthma have found that the age at which parents allow children to assume responsibility for taking their own medication may reflect the parents’ ability to supervise the child, not just the child’s maturity, and may result in unfounded confidence in the child’s ability.^13^ Adherence is sub-optimal in children whose parents have a more negative perception of asthma and doubt the necessity for inhaled steroids and are concerned about side effects.^13^

Guidelines such as the British Thoracic Society/Scottish Intercollegiate Guidelines Network (BTS -SIGN: 2016, SIGN 153)^2, 13^ and Global Initiative for Asthma (GINA, 2017)^14^ detail pathways of care for clinicians to use with children with asthma. They include written and personalised action plans and instruction on inhaler technique. The BTS–SIGN guidelines state that all people with asthma (and/or their parents or carers) should be offered self-management education, which should include a written personalised asthma action plan and be supported by regular professional review.^13, 15^

It is considered that families’ experiences of treatment are particularly important in understanding how adherence with asthma medication can be optimised by health professionals.

However, it not known to what extent young children are aware of asthma symptoms and their management or how health professionals approach children and parents in the clinical consultation. Indeed, to date there have been no qualitative studies that have explored the management of childhood asthma from the perspective of children, parents and health professionals. Therefore, the aim of this multi-perspective qualitative study was to explore the views of children, parents and health professionals to gain insight into the approach to clinical care in the management of childhood asthma.

## Results

Interviews were conducted with 13 Health Professionals: five General Practitioners (GPs) (one male, four female), four practice nurses (all -female), two secondary care consultants (one male, one female) and, two secondary care nurse specialists—both female). Two of the GPs were respiratory leads and there was one advanced nurse practitioner with a special interest in childhood asthma. Interviews were also conducted with nine parent–child dyads which included the presence of either one or both parents (two fathers, seven mothers, five sons, four daughters). The children were aged between 6–8 years.

All interviews were conducted between September 2015–March 2016. The interviews with primary care professionals were of 21 and 58 min duration, and the interviews with secondary care professionals were between 19 and 56 min duration. The duration of interviews with children was 6–18 min. The duration of interviews with parents was 14–32 min. Three key themes emerged from the analysis that were common to all data sets (practitioners, parents, and children): (1) child and parent awareness of symptoms, (2) management and child wellbeing, and (3) professional communication, education and consultation with families. Data to illustrate these themes are reproduced below.

### Child and parent awareness of symptoms

In general children and parents demonstrated a good awareness of the triggers of asthma and that symptoms were potentially controllable condition with appropriate treatment.

Children described how they become aware of the onset of symptoms of asthma using terms such as ‘wheezy’ and ‘breathless’;

*I cough and I get wheezy. It gets me very, very… not make me feel better. It hurts*. (C005, Male, 6 Years)

*Because I start to get breathless and I can’t breathe as well as I always do*. (C009, Female, 6 *Years)*

However, accounts from parents indicated that they struggled to distinguish between symptoms of asthma or breathlessness when their child was being physical active;

*It’s hard to know what’s general, “I’ve run too much and I’m puffed out,” or how much is asthma, I think. I don’t know myself, to be honest*. (P008, Mother of son, 6 years)

*He’s seven now and he’s aware, at sports, he’ll grab his puffer out of his boot bag. If I’m not there, he knows what to do*—*sit down, calm down, have his puffer. So he is aware of something*. (P001, Father of son, 7 years)

Health professionals believed that parents could foster a culture of over-protectiveness which could be a challenge to optimal management.

*…..you have parents who are almost over-reporting symptomology, and are, very perhaps they have had the label of asthma applied*—*and the parent is very restrictive because of asthma, which is probably contrary to the optimum approach…*.(HP001. Secondary Care, Consultant)

However, parents reported that their children had a good awareness of asthma and showed responsibility in alerting them to exacerbation of symptoms;

*I personally think he can build and strengthen not only his lungs, but his muscles and his awareness of his own asthma because I’m not going to be there forever. For his age, he’s quite clever. He’ll come to me and go, “Mummy, I need my inhaler.” I’m like, “Why?”, “Listen,” and he’ll make me listen to how he’s breathing*. (P003, Mother of son, 7 years)

Some GPs felt that children were not likely to express concerns and will ‘normalise’ any asthma related symptoms they may have and it is the parents who voice concerns.

*I don’t think the children express concerns, I think it’s parents that express concerns. I think if children are breathless, wheezing and coughing they consider that normal that’s what- I don’t think they recognise that is abnormal. I think children just keep on going don’t they? No, it’s the parents that express the concerns*…(HP008, Female GP)

### Management and child well-being

Most parents reported that their children were regularly absent from school following exacerbation of asthma symptoms, particularly in autumn and winter months;

*It has been difficult since she went to school. She doesn’t tend to, like I was trying to tell the paediatrician, she doesn’t tend to do a full term at school. So a term’s sort of six weeks. She tends to have a period of sickness almost every six weeks with asthma. It does vary, yes. Winter time, it is definitely worse* (P009, Mother of daughter, 6 years)

Children also reported that they did not like to miss school as it was felt to be disruptive both socially and with regard to continuity in learning.

*Well it is very bad and I don’t like it. Because I keep missing time off school and I want to join in with school. I just have to do my work even though I don’t know what we are learning about because we might be learning about something different*. (C009, Female, 6 years)

Furthermore, children experiencing symptoms of asthma not only missed school but tended to disengage from others.

*I behave like when you are like sick. All I do is like get a blanket and go on that sofa and put my arms under it and watch telly. Then if I am not like that I just go upstairs, go on my laptop …*..(C002, Male, 7 years)

Health professionals also suggested that acute exacerbations of symptoms occurred following withdrawal from using preventative inhaler due to parents thinking their child was well and because they did not understand the prophylactic role of the medication;

*It’s surprising how many people don’t know that or don’t respond in that way because what happens is that they take the preventive and see their child is fine and think they don’t need it anymore and then stop it. So why would you take a drug if you feel well, if you didn’t realise the prophylactic benefit of it?* (HP003, Female, Secondary Care, Nurse Specialist)

Some parents also expressed that they were unclear with regard to the role of medication as an intervention to relieve acute asthma symptoms or preventing future symptoms;

*So I’m a bit suspicious of whether he’s having the medicine for him now, or whether he’s having it to prevent something happening long-term*. (P006, Mother of son, 6 years)

The child in question described how he felt when taking his asthma medication through the inhaler and his perceived role of each inhaler;

*I stop and then I wait and then I wait until it is all up there. Then I breathe out. When I need to breathe, and there is a lot and I cannot breathe. One of them is for your breathing, one of them is for your chest*. (C006, Male, 7 Years)

Health professionals stated that children may normalise symptoms of asthma due to perceived stigma attributed to taking inhaler based medication. There was a suggestion that this lead to the child’s symptoms worsening; something which practitioners felt parents noticed but not the child themselves;

*The children just take it all in their stride really, more so than adults would. They don’t want to be different from other children I think. That’s what it is. They don’t want to be seen taking their inhaler at school. It’s not quite such a stigma these days, but there again, they’ve got to go and ask for their inhaler*. (HP005, Practice Nurse, Primary care, Female)

### Professional communication, education and consultations with families

Health professionals referred to clinical guidelines for treating childhood asthma, such as National Institute for Health and Clinical Excellence (NICE) and British Thoracic Society (BTS).

Only one GP mentioned guidelines that were developed in local secondary care/Clinical Commissioning Group. Clinical guidelines were not mentioned by parents;

*I think we do have a tendency to focus on the medical aspects of it, so the medication, the asthma reviews, the annual checks. The seeing them within 48 h, or reviewing within 48 h, if they’ve had an acute exacerbation. Following the guidelines. Following the step-up step-down treatment regimes. There are some NICE guidelines on it, and there are some local children’s hospital things as well in the protocol*. (HP010, GP, Female)

Another health professional stated that their role was to focus their attention on inhaler technique and asthma management plans and that symptoms should not occur if properly controlled.

*We’re supposed to focus on inhaler technique and whether they’re using inhalers, what they would do if things weren’t controlled, so management plans, I Focusing on what sort of things they can and can’t do because of their asthma, and recognising that they shouldn’t really have any symptoms if things are properly controlled*. (HP006, GP, Asthma Lead, Male)

Health professionals in secondary care suggested that consultations with children in primary care can be challenging with regard to providing education due to the time limitations or availability of specialist professionals.

…*.to be fair, in primary care, they’ve had perhaps 10 min of a GP. They’ve had-er, a practice nurse that’s not paediatrically trained, or we’re getting- so again, you can see why it’s happened*… (HP002, Nurse Specialist, Seconday Care, Female)

This view was further substantiated by health professionals, who felt that families do not have a clear understanding of the purpose and administration of their child’s medication;

*I think what happens is, GPs prescribe medications, and they don’t tell people what it does. I saw a child yesterday on intensive care who had two inhalers; a blue one and a brown one. They didn’t know the difference between the two, so when the child is unwell, they take both, but that’s just pointless*. (HP003, Nurse Practitioner, Secondary Care, Female)

…..

However, one Nurse Practitioner commented that they continuously reiterate to parents the purpose of medication;

*It’s just re-enforcing how you need to manage the medication and relate it to how the child is*. (HP004, Advanced Nurse Practitioner, Primary Care, Female)

Health professionals were also aware of concordance issues between children and parents and that parents may need prompting to consider the implications of handing over responsibility to the child.

*There’s a concordance issue with the children and there’s a concordance thing with the parents as well and that encouragement. guess they might want to hand things over to their children but they need to be able to know what the children are doing and what they’re doing and why they need to be doing it*. (HP004, Female, Primary Care, Advanced Nurse Practitioner)

However, GPs most were likely to see a child when symptoms are acute and there is not time or is deemed inappropriate to discuss management and thus is left to Nurse Practitioners.

*… we see them when they’re sick and so actually, the priorities are different and if you start talking about it, they might think “What are you going on about?” you know “Is my child okay? They’re having temperatures of 40. They’re wheezy”.So I think as much as much as I’d love to, in the ten minute appointment and often it’s on an on-call day that you see them, you don’t actually have that much opportunity*. (HP013, Female, GP)

Health professionals in secondary care expressed an awareness of the challenges faced by primary care professionals with regard to concordance and exacerbation of symptoms in children.

*…they’re Stepwise approach-is, step one to five. And we tend to see the ones that haven’t been as well controlled to step one and two. So primary care have actually referred us those. -I say more severe, but harder to manage individuals. And sometimes, they’re harder to manage, because in fact, there’s been concordance issues, perhaps, you know, there’s been other issues and whatever…*(HP001, Male, Consultant, Secondary Care)

A further component of the communication theme was a raised awareness of including the child in the clinical consultation. Indeed, health professionals felt that it was important to talk to both child and parent in their consultations but was deemed important to talk to child first to get a measure of self-reported adherence.

*I would aim to talk to the child, but I think the parent will often intervene. You talk to both, really, but start by talking to the child, and probably then go over to the parent. What we’re trying to ask them is do they know what they’re taking. “ If you ask the parent, you might get a different answer. So if you get to the child first, they’ll- and they can’t remember what they’re taking, that’s a good indication that they’re not taking it. Whereas the parent will probably say, “He has it twice a day.” So it’s the useful indicator*. (HP003, Female, Nurse Specialist, Secondary Care)

One GP felt that it was more appropriate to address the parents before addressing the child in the consultation as children may be daunted by talking to a GP.

*I would normally start talking to the parents and ask them about it. Then I would usually make an effort to actually ask the child what they thought, I think. Some children are a bit suspicious and worried about actually having to converse with the doctor, aren’t they, at that age? They find us all a bit scary*. (HP007, Female, GP, Asthma research lead)

With regard to imparting specific information, for example, with regard to inhaler technique, health professionals believed it was important to talk to both child and parent.

*Both, both, both (child and parent), and I think it’s really important, because I sort of, erm- I feel passionately that the kids are like sponges….. We go back to basic; check are they using their inhalers properly, and how are they using it? Is it consistent or whatever? So they’ll get inhaler technique, and we do that on every, visit. They’ll get action plans*. (HP002, Female, Nurse Specialist, Secondary Care)

## Discussion

### Main findings

The aim of this multi-perspective qualitative study was to explore the views of children, parents and health professionals to gain insight into the approach to clinical care in the management of childhood asthma. Three key themes emerged from the analysis; (1) child and parent awareness of symptoms, (2) management and child wellbeing, (3) professional communication, education and consultation with families.

Although parents were aware of exacerbation of asthma symptoms they were not always clear what triggered these exacerbations. They also struggled to distinguish between breathlessness and symptoms of asthma when their child was physically active. Nonetheless, some parents reported that their children had a good awareness of asthma and were responsible enough to access and take preventative medication as necessary. This view was supported by children’s descriptions of asthma symptoms, and their willingness to take medication. However, although there were rich data relating to adherence with inhaler-based medication there was little data explicitly relating to technique in the child and parent data sets.

Health professionals from both primary and secondary care reported that the control of asthma symptoms could be optimised through sustained adherence to preventer medication. Furthermore, health professionals were aware that families were often not clear with regard to the purpose and administration of different inhalers and that optimal adherence was rarely achieved. This was considered to be the main cause of exacerbations of asthma in children. A further prevalent view amongst health professionals was that in the absence of symptoms, parents became complacent towards supervising their child’s preventer treatment and symptoms then follow.

### Interpretation of findings in relation to previously published work

There was also data from both professionals and families that suggests there were mis-conceptions with regard to the use and benefit of asthma medication, particularly adherence with preventer medication. One explanation for sub-optimal adherence is that children’s and parents’ lay beliefs about asthma are at odds with the ‘biomedical’ model of asthma. The biomedical model presents asthma as an acute and chronic disease that can be controlled with proper management. However, parents may view asthma as a disease that is episodic, acute and uncontrollable, or that management is context dependent.^16^ The apparent non-alignment of views has implications for illness representation, treatment expectations and management decisions.^16–18^ Furthermore, if asthma is viewed as uncontrollable, it would be anticipated that more frequent exacerbations may be perceived as acceptable. Symptom recognition may also be highly variable between parents and contrast with asthma symptoms that are conventionally recognised by the medical community.^16, 17, 19^ Children may have a greater awareness of asthma symptoms and for adherence with asthma medication than health professionals, teachers and parents currently acknowledge. That said, data from the professionals working in secondary care suggested that they are more likely to treat children who demonstrate poorer knowledge and adherence with medication. The secondary care professionals were also aware that the care of children in primary care may be compromised due to lack of time and/or the availability of paediatrically trained professionals.

### Implications for future research, policy and practice

Health professionals’ awareness of child and parent perceptions of asthma can influence asthma management decisions and the provision of information for families. Other recent qualitative research has highlighted a need for continuous patient education interventions for individuals with asthma.^20^ Moreover, there have been attempts to consolidate the informational needs of parents with regard to the management of childhood asthma. Indeed, a recent review of the information needs of North American parents of children with asthma^21^ identified a needs inventory comprising; asthma basics (physiology, symptom recognition); treatment modalities (short-term/long-term medication, mechanism of action); coping (emotions, worry, developing an action plan) and medical expectations (when to seek medical care). The four parental information needs identified in this review provide a preliminary inventory of parental needs with which to integrate evidence-based information for educational purposes and future interventions studies.

Finally, the present findings suggest that future research may benefit if there was a focus on the engagement of children and parents in asthma consultations with a view to developing interventions aimed at reinforcing messages with regard to appropriate and optimal inhaler use.

### Strengths and limitations of this study

Interviewing children, parents and health professionals meant we were able to consider management of childhood asthma from the perspective of all those involved and serves to increase the uniqueness of the study. Furthermore, as the interviewer and primary analyst (A.S.) was not a member of a clinical team serves to enhance the robustness of the findings. While we sampled primary care practitioners and families from practices based in the South West of England they were all located in areas of low to middle socio–economic status. Thus the findings may not be generalised to families of higher socio–economic status. The representativeness of the health professionals is also a potential limitation due to the dispersion of roles within the sample (i.e., Consultant, GP, Nurse) and therefore it cannot be assumed that data saturation was achieved in these sub groups. A further limitation is that the children struggled to focus on the nature of interview questions and sometimes needed prompting by parents.

## Conclusion

The present findings have implications for the delivery of childhood asthma services and health professionals’ interaction with families. Thus health professionals and parents may help to optimise the management of childhood asthma if children are more engaged in their asthma consultations. There is also a need for intervention to help parents optimise the management of childhood asthma with the focus on recognition of symptoms and adherence with treatment. Thus, GPs and nurse practitioners could emphasise asthma management plans in their consultations with children and encourage joint management strategies amongst children and parents and in so doing reduce the burden of symptoms for children with asthma and their families.

## Methods

### Interviews

Semi-structured interviews were conducted in the course of qualitative research exploring children’s, parents and health professionals’ views of managing childhood asthma and attitudes towards engaging in physical activity.^22^

The research was based in South-West England and participant recruitment was facilitated by the Clinical Research Network (CRN). Seven primary care practices were identified by the CRN.

Children currently being treated for asthma at primary care practices in the South West of England were approached for participation in the study. These practices were all located in areas of low to middle socio–economic status. The practices were asked to post invitation letters, information sheets and pre-paid reply envelopes to the parents of these children informing them about the study. If the parents were and/or their child was willing to be interviewed returned the reply slip to the first author (A.S.). A total of 79 invitation letters and information sheets were sent, with reminders sent 3 weeks later (see Table 1). Responses were received from ten families and interviews were held with nine families. One family decided not to participate once the researcher had contacted them to arrange an interview.

**Table 1:** Family recruitment letters/response per practice

Practice location	Letters sent	Reminder letters	Total response
Practice 1, West	16	14	2
Practice 2, South	20	19	2
Practice 2, South	21	21	4
Practice 4, Central	22	22	1
Total	79	76	9

GPs and practice nurses were recruited through the same primary care practices as families.

GPs and nurses involved in managing childhood asthma in each of the four practices were invited to participate in an interview.

Recruitment of secondary care health professionals was facilitated by a respiratory consultant (J.H.) who provided A.S. with names of health professionals treating children with asthma. J.H. was not made aware of who was interviewed.

### Topic guides

Topic guides were developed by A.S., K.T. and R.J. to facilitate the interviews children, parents and health professionals (Appendix 1, 2 and 3). The topic guides included items on experience of childhood asthma, symptoms and management. The topic guides also include items regarding attitudes towards and engagement in physical activity although beyond the scope of the present article. The topic guides were piloted during the initial two interviews and then small revisions made. These ‘pilot’ interviews were included in the analysis. The guides were developed in parallel and all covered key areas to aid triangulation of children’s parents’ and professionals’ accounts.

Interviews with children, parents, and health professionals were conducted on a face-to-face basis by A.S., an experienced qualitative researcher. Children and parents were interviewed in their homes. Primary care practitioners were interviewed in their practice rooms and secondary care practitioners were interviewed in a University/Health Trust research and education facility.

Parents and health professionals provided written consent prior being interviewed, and parents also provided written consent for their children to be interviewed. No feedback was given to the health professionals or parents following the interviews, in terms of receiving participant feedback on the initial analysis. However, throughout each interview, the researcher summarised findings and checked with the interviewee he had correctly understood what had been said.

### Data analysis

All the interviews were audio-recorded and transcribed verbatim. Transcripts of each data set (two children, two parents, two primary care, and two secondary care) were read and re-read by two experienced qualitative researcher/data analysts (A.S. and K.T.) to familiarise themselves with the data. The analysts then met to discuss their overall impressions of each data set and analysis approach. It was agreed that all the data sets should be analysed thematically as this would enable comparisons to be made both within and across the three data sets, i.e., children, parents and health professionals.^23^ The analysts A.S. and K.T. independently coded the sampled transcripts and then met to discuss their coding and to develop coding frames for each interview data set. Next, A.S. and K.T. coded transcripts using an agreed coding frame and met again to verify coding. Once each coding frame was finalised, all the transcripts were imported into NVivo (Version 10)^24^ and electronically coded. Each data set was analysed separately before comparisons were made between the accounts of each interview group.

All relevant data pertaining to the transcribed interviews are available from the authors.

## Electronic supplementary material

Appendices
