# Supplementary material for: Children’s, parents’ and health professionals’ views on the management of childhood asthma: a qualitative study
Source: NPJ Prim Care Respir Med. 2017 Sep 11;27:53. doi: 10.1038/s41533-017-0053-7 (PMC5593954; doi:10.1038/s41533-017-0053-7)
Supplement: Supplementary file 1 — Appendices [file 41533_2017_53_MOESM1_ESM.docx]

**Appendix 1**

**Topic Guide: Children**

**Background**

Tell me a bit about yourself, for example, what do you like doing?

**Knowledge and treatment of Asthma**

How long have you had asthma?

How do you manage your asthma?

What treatment do you take, what things do you avoid doing?

Do your friends know you have asthma?

Do you know anyone else your age with asthma?

How does your asthma affect you/make you feel?

Prompt for: Physical and mental effects

Do you worry about your asthma?

If you do worry, why do you worry?

- If not, why not?

What do you worry might happen?

Have you ever had an asthma attack?

If so, thinking about the last attack you had, why do you think it happened? What caused it?

Does it stop you doing anything?

Prompt for:

- running around
- cycling your bike
- riding your scooter
- swimming
- feeling like you can join in with your friends
- going out with friends and staying over at friend’s houses

How do you feel about it stopping you from doing these things?

Would you want to be more physically active?

**Views of physical activity**

We are interested in physical activity. Physical activity is when you move around like running, playing, skipping, swimming or riding your bike.

Are you ever active in the ways I have just described? Are there any other ways in which you are active?

When are you active?

- at home
- at school in PE or in the playground
- at the weekends

How often are you physical active?

- Most days of week?
- A few days?
- Not that often?

When you are active, do you hold back or just go for it?

If they hold back, ask why, what they worry about and what might help them worry less.

Do you like being active. Why/why not?

If you didn’t have asthma, would you behave any differently?

If so, in what ways?

Do you feel your parents or teachers worry about you being active?

Have your parents or teachers ever stopped you from doing physical activity because you have asthma?

Does anybody encourage you to be active?

If yes: Who

- Parents
- Teachers
- Friends /other children

If we asked you to do more physical activity, what activities would you want to do?

What help would you need to do it?

Who would you like to help you?

How would you like your mum or dad to help you be more active?

How could they encourage you to be more active?

How would you feel about them doing that?

Is there anything else you would like to say about asthma and physical activity?

**Appendix 2**

**Topic Guide: Parents**

**Background**

Just to provide some context, please can you tell me a little bit about yourself and your family?,

- .e.g number of children, their ages, what you do.

Knowledge of Asthma

Tell me a bit about your child’s asthma:

When did it start?

How does it affect him/her?

How is it managed?

How often does s/he need to use an inhaler?

What do you think caused it initially?

What do you think triggers it now?

Is there anything you think your child cannot do because of his/her asthma?

In what ways do you treat him/her differently because of his/her asthma?

If someone else is looking after him/her, do you mention their asthma to them?

If so, what do you tell them?

**Views of Physical Activity**

We will now focus on physical activity and particularly moderate to vigorous intensity physical activity, which is when you get slightly hot and sweaty and a little out of breath

Do you think your child would benefit from being more physically active? Prompt for rationale behind the answers given. Prompt for how they think it would benefit their child in general and in relation to their asthma

Do you worry about your child being physically active or do you just treat them the same as if they did not have asthma?

What physical activity do you do as a family?

What physical activity does your child do, both in and out of school and at the weekends?

Has anyone advised you to encourage your child to be more physical active?

Prompt for:

- Teacher
- Health professional

Do you encourage your child to be physically active?

Has your child ever been excused from doing physical activity at school because of their asthma?

If so: why was your child excused? How did you feel about this?

What prevents and helps your child from being physically active?

How does their asthma limit what they can do in terms of;

- Type of activity?

Is there anything else you would like to say about your child, asthma and physical activity?

**Appendix 3**

**Topic Guide: Primary / Secondary Care Practitioners**

**Background**

To start could introduce yourself and your clinical role?

How long have you worked at this clinic / respiratory medicine?

How often to you see children with asthma?

**Consultations with children with asthma**

What do you see as the main aims of the treatment/management you provide?

What are you trying to achieve when managing childhood asthma?

What care or advice do you normally give to parents?

We are interested in children aged 6 to 8 years. Is there anything we need to particularly think about, when thinking about management in this age group?

What do you think are the main concerns parents of children aged 6-8 have about the management of their child’s asthma?

What do you think are the main concerns of the child?

How do you find working with these families?

- Are they motivated to change and manage their child’s condition?
- Are they actively involved in the management?
- Do they attend follow-up appointments etc?
- Do you talk mainly to the parent or do you also involve the child?

**Family Guidance**

What information do families with young children with asthma currently receive?

Do you tell parents to encourage their children to engage in physical activity and reduce screen viewing? If so, why? If not, why not?

How do they respond to this advice (positively or reluctantly)?

What type of questions / concerns do they have?

How does the child respond?

What factors do you think support or hinder parents in increasing their child’s physical activity levels?

Prompt for:

- Time
- Money
- Parental concerns
- Asthma attacks

What barriers do you think they might face when trying to reduce their child’s screen viewing time?

What might help them achieve this behavioural change?

**Clinical guidance and physical activity**

What are your views about the use of physical activity in the management of childhood asthma? Prompt for pros and cons, potential effectiveness, concerns or issues practitioners might be worried about.

What type / intensity of physical activity do you think young (6-8 year olds) children should engage in?

Prompt for:

- How often should they engage in physical activity?
- Intensity?
- Duration?
- Frequency?
- Mode?

How would the advice you give to a child with asthma, about physical activity, be different or the same as the advice you would give to a child without asthma?

Are there any reasons or situations when you would not advise a child with asthma to engage in physical activity?

What do you see as the barriers to activity for children with asthma?

Are there any comments you would like to make in general, which we have not covered?
